# Humor norms for 4,997 English words

**DOI:** 10.3758/s13428-017-0930-6

**Published:** 2017-07-14

**Authors:** Tomas Engelthaler, Thomas T. Hills

**Affiliations:** Department of Psychology, University of Warwick, Gibbet Hill Road, Coventry, CV47AL UK

**Keywords:** Humor, Crowd-sourcing, Ratings, Gender differences

## Abstract

Humor ratings are provided for 4,997 English words collected from 821 participants using an online crowd-sourcing platform. Each participant rated 211 words on a scale from 1 (*humorless*) to 5 (*humorous*). To provide for comparisons across norms, words were chosen from a set common to a number of previously collected norms (e.g., arousal, valence, dominance, concreteness, age of acquisition, and reaction time). The complete dataset provides researchers with a list of humor ratings and includes information on gender, age, and educational differences. Results of analyses show that the ratings have reliability on a par with previous ratings and are not well predicted by existing norms.

## Introduction

The appreciation of humor is a fundamental, albeit mysterious, part of human cognition. We laugh at things like *Monty Python* and the work of Douglas Adams, but find topics like mass shootings and the Holocaust off limits. Other topics, like sunsets and freedom, may lie somewhere in between. What makes one thing funnier than another? And what makes some topics inviolable in relation to humor? To help develop this research, we provide the first set of humor norms for a large collection of 4,997 common words. The aim of providing this data is to help enrich the resources available for understanding the cognitive, developmental, and applied aspects of humor.

Humor has a long history of theoretical investigation. Darwin (1872) called humor “tickling the mind.” Thomas Hobbes (1840) referred to it as a feeling of “sudden glory.” These represent a selection from a long list of efforts to provide a theory of humor (reviewed in Hurley, Dennett, & Adams, 2011; Keith-Spiegel, 1972; Wyer & Collins, 1992). These include biological theories – such as the Darwin-Hecker hypothesis that humor is a cognitive analogue of physical tickling (Fridlund & Loftis, 1990; Harris & Christenfeld, 1997); superiority theories, such as Hobbes notion of “sudden glory” over another individual or one’s previous self (Hobbes, 1840); release theories, such as that proposed by Spencer (1860) and later Freud (1928), that humor is a means of reducing excessive arousal; incongruity-resolution theories (Shultz, 1976; Suls, 1972), perhaps first noted by Kant (1790/1914), in his observation that “In everything that is to excite a lively convulsive laugh there must be something absurd,” and later developed by Schopenhauer (for an overview, see Roeckelein, 2006), who suggested the “ludicrous” required a “contrast…between representation of perception and abstract representations.” Still further theories have focused on the adaptive value of humor as an error correction mechanism and faulty logic detection system (Minsky, 1981), most recently and thoroughly developed by Hurley, Dennett, and Adams (2011). A similar version of this theory has been called the benign violation theory (McGraw & Warren, 2010), which suggests a person must realize the stimuli is incongruous with their expectations (violation), but also that this incongruity is not harmful given the context (benign).

The onslaught of theories aimed at understanding humor reflects our common experience that humor is a key ingredient in what it means to be a healthy human. It may even be uniquely human and, continuing the noble history validating intuition with Latin, Koestler (1964) referred to humans as *Homo ridens*, “laughing man” (see also Milner, 1972). Whether or not it is unique to humans, humor has well-documented influences on well-being and health, including self-concept, coping with stress, and positive affect (Cann & Collette, 2014; Galloway & Cropley, 1999; Martin et al., 1993; Mora-Ripoll, 2011). Humor research also contains a wide body of literature concerned with understanding adult and child personality development (Martin, 1998; McGhee, 1971) and gender differences (Abel, & Flick, 2012; Hay, 1995; Mickes, Walker, Parris, Mankoff, & Christenfeld, 2012). The latter associated with the evolutionary hypothesis that humor plays a role in male mating displays (McGee & Shevlin, 2009), and which is further supported by gender differences in response to humor in the brain (Azim, Mobbs, Jo, Menon, & Reiss, 2005; see also Goel & Dolan, 2001).

In addition, cracking the riddle of what makes things funny has also been the motivation for a number computational algorithms designed to create humor, such as JAPE (Binsted, Pain, & Ritchie, 1997), STANDUP (Manurung et al., 2008), WISCRAIC (McKay, 2002), and HAHAcronym (Stock & Strapparava, 2003), as well as algorithms to detect and classify humor (Davidov, Tsur, & Rappoport, 2010; Mihalcea & Strapparava, 2005).

Much of the theory and empirical work briefly outlined above focuses on complete multi-word jokes, such as this zinger by Steven Wright: “I couldn’t repair your brakes, so I made your horn louder.” To this end, a number of studies have taken to rating and creating databases of jokes in an effort to allow researchers disaggregate the various mechanisms that make them work (e.g., Goldberg, Roeder, Gupta, & Perkins, 2001; Wicker, Thorelli, Barron III, & Willis, 1981). A few studies have looked at single non-words (Westbury, Shaoul, Moroschan, & Ramscar, 2016), suggesting the absurdness of a non-word results in associated humor. None, to our knowledge, have focused on single English words.

The database we present here offers a basis for studying humor in perhaps a highly rudimentary “fruit fly” version, at the level of a single word. If single words have reliable humor ratings, they provide humor in miniature, allowing us to investigate humor in relation to the many existing lexical norms. These include some that are directly related to past theories – such as Freud’s (1928) arousal theory – and others that offer at least some insight into processing and expectation, such as reaction times and frequency.

The collection of the humor norms follows on previous work demonstrating the advantage of crowd-sourcing in psychological norm development: for example, Warriner, Kuperman, and Brysbaert (2013) have collected valence, arousal, and dominance ratings for 13,915 English words; Brysbaert, Warriner, and Kuperman (2014) collected concreteness ratings for nearly 40,000 English words; and Kuperman, Stadthagen-Gonzalez, and Brysbaert (2012), collected age of acquisition ratings for 30,000 English words. These were in turn based on the value of previous norms, such as the Affective Norms for English, provided by Bradley and Lang (1999).^1^ Still other normative ratings have investigated different word properties, which have provided the basis for further investigating their influence on cognition, such as imageability and familiarity (Stadthagen-Gonzalez & Davis, 2006), pleasantness (Bellezza, Greenwald, & Banaji, 1986), and meaningfulness (Paivio, Yuille, & Madigan, 1968).

These normative datasets have proven highly fruitful. For illustration, Dodds et al. (2015) used valence ratings to assess a universal positivity bias. Alhothali and Hoey (2015) used valence ratings to predict readers’ responses to news articles. And Hills and colleagues (Hills & Adelman, 2015; Hills, Adelman, & Noguchi, 2016) used concreteness, age of acquisition, and lexical reaction times to evaluate the changing history of American English over the last 200 hundred years.

Here, we provide a large dataset of single-word humor ratings along with the demographics of the raters. The list of rated words was formed from the intersection of overlapping previous non-humor word norms, allowing us to provide an analysis of how word-level humor relates to valence, arousal, word length, concreteness, word processing time and word frequency. Secondly, breaking down our dataset by demographics, we provide a separation of humor by gender.

## Methods

### Stimuli

The words in the norms are chosen from the intersection of the valence, arousal, and dominance norms (Warriner, Kuperman, & Brysbaert, 2013), age of acquisition norms (Kuperman, Stadthagen-Gonzalez, & Brysbaert, 2012), lexical decision norms (Keuleers, Lacey, Rastle, & Brysbaert, 2012), and frequency norms (Van Heuven, Mandera, Keuleers, & Brysbaert, 2014). This resulted in 7,775 words, from which the final word list of 5,000 words was randomly sampled. This reduction in list size increases the number of raters exposed to a single word, given a fixed number of participants.

Participants provide information in response to demographic questions (age, gender, language, country growing up, and education), the humor rating of calibrator words, and the humor rating of 200 words randomly sampled from the pool of 5,000 words. The calibrator words are a list of 11 words that spanned the range of humor rating in a pilot study (with 150 participants and 500 randomly sampled words). The calibrator words are presented in Table 1. Following previous studies (e.g., Brysbaert et al., 2014; Warriner et al., 2013), participants saw the calibrator words first, with the aim of showing the participant the range of the humor scale and increasing the reliability of subsequent ratings. The calibrator words were followed by the random sample of 200 words. The word sample was different for each participant, generated in real time when the participant opened the online questionnaire.

**Table 1:** Calibrator words presented to participants

Word	*Mean humor rating (Pilot)*
Drought	1.13
Deathbed	1.55
Cleaver	1.69
Oxide	1.8
Rainstorm	1.91
Lurch	2
Maroon	2.08
Driftwood	2.23
Cleat	2.4
Walnut	2.67
Turd	3.78

### Data collection and participants

Participants were recruited using Amazon Mechanical Turk. Any registered member of Amazon Mechanical Turk was allowed to participate, with the requirement of fully completing the study (partial data was not recorded), and only doing the study once. Upon accepting the study, the participant was redirected to a website that delivered the instructions and words for rating. The introduction read as follows:

You will rate how you felt while reading each word. There will be approximately 200 words. The rating scale ranges from 1 (humorless = not funny at all) to 5 (humorous = most funny). At one extreme of the scale, you find the word dull or unfunny; in that case, you should give the word a rating of 1. At the other extreme of the scale, you feel the word is amusing or likely to be associated with humorous thought or language (for example, it is absurd, amusing, hilarious, playful, silly, whimsical, or laughable); in this case, you should give the word a rating of 5. The scale also allows you to describe intermediate of humor; if you feel the word is neutral (neither humorous nor humorless), select the middle of the scale (rating 3).

After you fill out some basic information about yourself, a word list will appear. Simply click the most accurate humor rating for each word. Once you finish rating the words, we will ask you a couple of questions about the way you use humor. Please work at a rapid pace and don't spend too much time thinking about each word. Rather, make your ratings based on your first and immediate reaction as you read each word.

The introduction was followed by the list of 211 words, each word having five buttons presented just below it, numbered from 1 to 5, with the extremes labeled “humorless” (1) and “humorous” (5). The first 11 words were the calibrator words. The combination of the remaining 200 words was different across participants. After selecting a rating for a word, the word disappeared from the list. Upon rating all words, the participant could press the “Submit” button. The participant was then presented with a debrief page and directed back to Amazon. Each participant was paid US$1. The study took approximately 15 min to complete, including reading the instructions and the debrief page.

## Results

### Data trimming

The data were presented to 950 participants. 102 participants were removed due to incomplete submissions, errors in the data and improperly submitting their responses. Five participants were removed due to low variability of their responses (the standard deviation of their humor ratings, on a 1–5 scale, was smaller than 0.2, indicating they chose roughly the same value for all words). Twenty-two participants were removed because they indicated their primary language was not English. The final data consisted of 821 participants. The raw data had 173,231 individual data points, referring to a single rating of a single word. Ratings were collected for 4,997 words, with each word rated by at least 15 participants. The average number of participants rating a word was 33 (*M* = 32.93, *SD* = 5.64, *n* = 4986). The 11 calibrators were rated by all 821 participants.

### Demographics

Participants identified as female in 478 cases (58%), as male in 341 cases (42%), and two participants chose not to answer (<1%). The mean age of participants was 35 years (*M* = 35.37, *SD* = 11.74, *n* = 821), ranging from 18 to 78 years. Table 2 presents the education demographics.

**Table 2:** Education distribution of the participants

Education type	*Number of participants*	*% of participants*
Elementary School	5	<1%
Some High School	5	<1%
High School Diploma	235	29%
Undergraduate Degree	434	53%
Postgraduate Degree	126	15%
Higher than Postgraduate Degree	16	2%

### Humor ratings

For each word, all of the humor ratings were summed and divided by the number of participants rating the word. This resulted in a Mean Humor Rating (MHR) of each word. The split-half reliability of the individual ratings was 0.64, slightly lower than previously collected for arousal ratings (0.69 in Warriner, Kuperman, & Brysbaert, 2013). This suggests there are considerable individual differences, which may be of interest for future research. The MHR for each word is provided in the supplementary material. MHR were also computed for each gender separately. Table 3 and 4 shows the descriptive statistics of MHR across all participants.

**Table 3:** Descriptive statistics of mean humor ratings (MHR)

Statistic	*Value*
Mean	2.41
Standard deviation	0.44
Median	2.34
Minimum	1.18
Maximum	4.32
Skew	0.78
Kurtosis	0.87

**Table 4:** Words with the most extreme mean humor ratings

Positive extreme	*Negative extreme*
Booty (4.32)	Rape (1.18)
Tit (4.25)	Torture (1.26)
Booby (4.13)	Torment (1.3)
Hooter (4.13)	Gunshot (1.31)
Nitwit (4.03)	Death (1.32)
Twit (4)	Nightmare (1.33)
Waddle (4)	War (1.33)
Tinkle (3.94)	Trauma (1.35)
Bebop (3.93)	Rapist (1.37)
Egghead (3.92)	Distrust (1.38)
Ass (3.92)	Deathbed (1.39)
Twerp (3.92)	Pain (1.39)

**Fig. 1:**
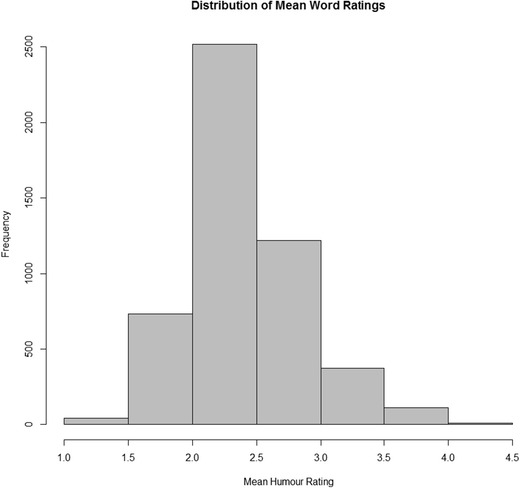
Distribution of mean humor ratings (MHR) across 4,997 English words. The distribution of MHR covers a range of 3.14 units. The most humorless word in the norms is “rape” (1.18) and the most humorous word is “booty” (4.32). Table 4 lists the 12 most extreme words at the end of the distribution

The MHR distribution was positively skewed, indicating that more words are rated as *humorless* than *humorous*. This is in contrast to previously collected valence norms, which tend to be negatively skewed. People have an intrinsic positive bias for valence, interpreting most words as positive (Dodds et al., 2015; Warinner et al., 2013). For humor, the opposite is true – most words are rated closer to humorless than humorous. The shape of the MHR distribution is shown in Fig. 1.

**Fig. 2:**
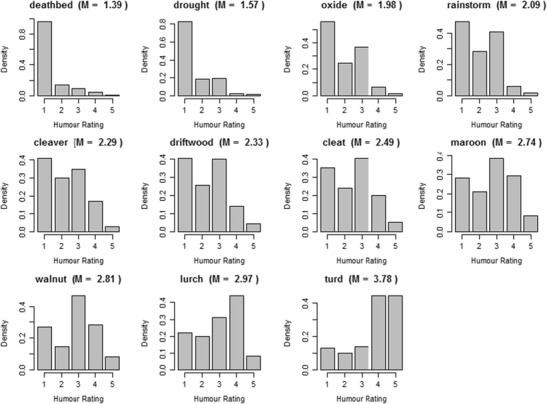
Distribution of ratings over all participants for each of the 11 calibrator words

The calibrator words were presented to all 821 participants. Their distributions were calculated individually. To provide an indication of how words across the distribution are rated by all of the participants, Fig. 2 presents the distributions for each of the calibrator words separately.

### Correlations

Table 5 shows the correlations of the MHR with other linguistic metrics available from existing norms. The strongest correlation is with frequency (British National Corpus), with less frequent words rated as more humorous. Words less frequent in SUBTLEX (movie subtitles) were also rated as more humorous. Words that are associated with longer reaction times in lexical decision tasks were also rated as more humorous.

**Table 5:** Correlations between 11 lexical measures

Variable		1	2	3	4	5	6	7	8	9	10	11
1	Mean Humor Rating											
2	Age of Acquisition	0.08										
3	Word Length	-0.06	0.26									
4	Frequency (BNC)	-0.42	-0.40	-0.26								
5	Frequency (SUBTLEX)	-0.30	-0.57	-0.33	0.78							
6	Lexicality RT	0.27	0.56	0.30	-0.71	-0.73						
7	Valence	0.09	-0.29	0.03	0.23	0.19	-0.22					
8	Arousal	0.05	0.07	0.05	-0.06	0.07	-0.04	-0.16				
9	Dominance	0.01	-0.22	0.00	0.23	0.18	-0.20	0.61	-0.15			
10	Concreteness	0.12	-0.35	-0.05	-0.11	0.00	-0.05	0.11	-0.18	0.05		
11	Frequency (ANC)	-0.40	-0.38	-0.27	0.88	0.78	-0.68	0.22	0.00	0.22	-0.15	

### Gender differences

The mean ratings for the two genders were identical (*M*_*M*_ = 2.41, *SD*_*M*_ = 0.51; *M*_*F*_ = 2.41, *SD*_*F*_ = 0.48; males and females rate the same number of words, *n* = 4,997). The male and female ratings are strongly correlated, *r*(4,995) = .60, *p* < .001. There are, however, gender differences in the ratings of individual words. Table 6 shows words with the biggest disagreement between genders.

**Table 6:** Words with the largest differences between male and female ratings

Words rated more humorous by males	*Words rated more humorous by females*
Bondage (1.55)	Giggle (-1.92)
Birthmark (1.47)	Beast (-1.61)
Orgy (1.47)	Circus (-1.6)
Brand (1.46)	Grand (-1.5)
Chauffeur (1.35)	Juju (-1.45)
Doze (1.34)	Humbug (-1.38)
Buzzard (1.34)	Slicker (-1.38)
Czar (1.30)	Sweat (-1.38)
Weld (1.29)	Ennui (-1.36)
Prod (1.27)	Holder (-1.35)
Corn (1.27)	Momma (-1.35)
Raccoon (1.26)	Sod (-1.35)

*Note*. Numbers in brackets are the difference in ratings between genders. They are computed as MHR_M_ – MHR_F_: a positive value means the word is rated as more humorous by males, a negative value means it was rated as more humorous by females

**Fig. 3:**
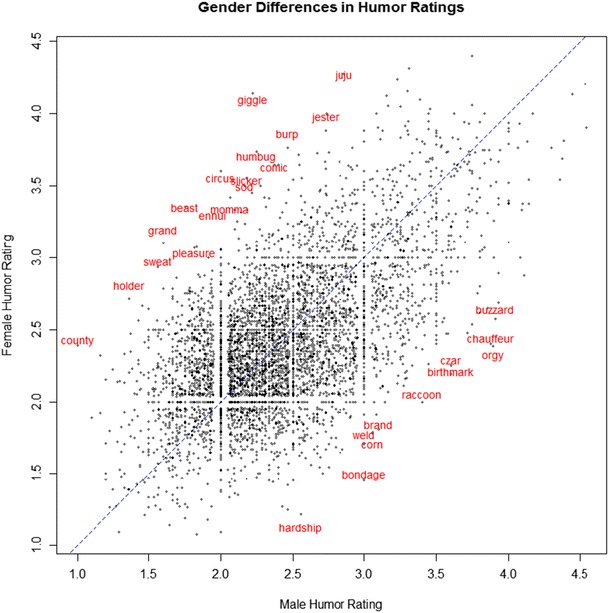
A plot of male and female mean humor ratings (MHR) for each of the 4,997 words. Words having an absolute gender difference larger than 1.25 are labeled in red. The blue line has an equation of y = x. Slight jittering was applied to the word labels to improve readability

The words of biggest disagreement are in essence the outliers of an MHR_M_ – MHR_F_ plot, where MHR_M_ is the mean humor rating of male participants and MHR_F_ is the mean humor rating of female participants. This relationship is shown in Fig. 3.

Similarly, it’s also possible to show words that males and females have high agreement on. For illustration, we restricted the data to 5% of the words with the lowest disagreement (*n* = 250). This subsample was then sorted by overall MHR (for both genders), resulting in a list of words that are perceived as humorous by both males and females (see Table 7). Note that the 5% subsampling criterion is arbitrary in this case. We encourage the reader to design and carry out their own, more comprehensive analyses using the dataset.

**Table 7:** Words with the lowest differences in gender, while scoring high on mean humor rating (MHR)

MHR	*Gender difference (MHR*_*M*_ *– MHR*_*F*_ *)*
Chug (3.73)	-0.01
Fluff (3.72)	0.02
Scrotum (3.68)	0.03
Jabber (3.65)	-0.00
Joke (3.64)	-0.03
Buttocks (3.63)	0.02
Boon (3.49)	0.02
Yank (3.32)	0.00
Tinker (3.31)	0.02
Prance (3.31)	0.00

*Note*. The values in the first column show the MHR for both males and females combined. The values in the second column show gender differences, computed as MHR_M_ – MHR_F_. The words represent the most humorous words in our dataset, which also have the absolute value of the gender difference smaller than .05

### Age differences

To allow for further investigation of age differences, we also provide the MHR for younger and older participants separately. The mean age of all participants was 35 years (*M* = 35.37, *SD* = 11.74, *n* = 821), with a median value of 32. The two groups (younger and older) were constructed as an outcome of a median split of the dataset. The younger group consists of participants with age ≤32 (n = 424, *M* = 26.7, *SD* = 3.52, min = 18, max = 32), the older group of participants with age >32 (n = 397, *M* = 44.7, *SD* = 10.2, min = 33, max = 78). The overall humor ratings of the younger participants (*M*_*Y*_ = 2.42, *SD*_*Y*_ = 0.49) were comparable to those of the older participants (*M*_*O*_ = 2.41, *SD*_*Y*_ = 0.48). The ratings of the younger and older groups are strongly correlated, *r*(4,995) = .63, *p* < .001.

In line with the gender analysis above, it is possible to list words of high disagreement between age groups (i.e. *M*_*Y* –_ *M*_*O*_; see Table 8).

**Table 8:** Words with the largest rating differences between younger and older participants

Words rated more humorous by younger	*Words rated more humorous by older*
Goatee (1.49)	Caddie (-1.56)
Reform (1.46)	Birthright (-1.45)
Joint (1.43)	Squint (-1.31)
Germ (1.39)	Jingle (-1.28)
Hunchback (1.34)	Burlesque (-1.28)
Frock (1.32)	Bulkhead (-1.27)
Rating (1.29)	Limey (-1.26)
Squaw (1.29)	Pixie (-1.26)
Filth (1.25)	Pong (-1.25)
Collie (1.23)	Willow (-1.23)
Squabble (1.19)	Housewife (-1.23)
Gangster (1.15)	Bathing (-1.23)

*Note*. Numbers in brackets are the difference in ratings between age groups. They are computed as MHR_Y_ – MHR_O_: a positive value means the word is rated as more humorous by younger participants, a negative value means it was rated as more humorous by older participants

The supplementary material contains age-separate ratings for each word, allowing for further analyses of age differences in humor ratings.

## Discussion

Using the ready availability of large online data collection, the present study has created a database of single-word humor ratings. The statistical analyses show that people view words as humorous to a varying extent, with a skew towards seeing the majority of words as humorless. The appraisal of single-word humor can be reliably measured across participants, similarly to that of arousal.

The present study shows examples of analyses that can be carried out with the humor dataset. Specifically, it is possible to show correlational relationships between humor rating and other variables (i.e., frequency and lexical reaction times). This approach may, in turn, inform us on how the underlying mechanisms of humor work, or at the very least, where to look in the future. Additionally, it is possible to investigate gender differences in humor appraisal.

Besides the above-mentioned examples, we identify three fields of interest for future research. First, using existing databases of jokes (e.g., Goldberg, Roeder, Gupta, & Perkins, 2001), the humor ratings make it possible to explore the relationship between the appraisal of humor on the joke level and on the single-word level. Second, the humor norms provide a resource for machine learning methods to establish the best predictors of word level humor, which can later be evaluated in psychological experiments. Third, individual ratings of words in relation to the norms can provide a basis for understanding individual differences in humor styles (e.g., Martin, Puhlik-Doris, Larsen, Gray, & Weir, 2003). Finally, like previous ratings, the humor norms may offer new insights into text analysis and the creation of psychological stimuli.

## Availability

The mean humor ratings are freely available as part of our dataset. The data can be accessed at *https*://*github.com*/*tomasengelthaler*/*HumorNorms*, downloadable as a.csv file. The sheet is organized alphabetically, by word label. It includes the mean humor rating for all participants combined (mean_ALL), along with the standard deviation (sd_ALL) and the number of participants rating a word (n_ALL). The same three variables are available exclusively for participants identifying as male (mean_M/sd_M/n_M) and for those identifying as female (mean_F/sd_F/n_F). Additionally, the variables are also presented according to the median split of age, dividing participants into a younger group (age ≤32; mean_young/sd_young/n_young) and an older group (age >32; mean_old/sd_old/n_old).

## Footnotes

1 Dutch (Moors et al., 2013), Finnish (Söderholm, Häyry, Laine, & Karrasch, 2013), French (Monnier, & Syssau, 2014), German (Kanske, & Kotz, 2010), Italian (Montefinese, Ambrosini, Fairfield, & Mammarella, 2014), Portuguese (Soares, Comesaña, Pinheiro, Simões, & Frade, 2012), and Spanish (Redondo, Fraga, Padrón, & Comesaña, 2007).
